# High Fibre Gluten-Free Fresh Pasta with Tiger Nut, Chickpea and Fenugreek: Technofunctional, Sensory and Nutritional Properties

**DOI:** 10.3390/foods9010011

**Published:** 2019-12-21

**Authors:** Beatriz Llavata, Ana Albors, M. Eugenia Martin-Esparza

**Affiliations:** Institute of Food Engineering for Development, Universitat Politècnica de València, Camino de Vera s/n, 46022 Valencia, Spain; beallaca@etsiamn.upv.es (B.L.); aalbors@tal.upv.es (A.A.)

**Keywords:** coeliac disease, in-vitro starch digestibility, legume flour, tiger nut flour, fibre, galactomannans

## Abstract

Gluten-free pasta production with a low glycaemic index and improved nutritional profile is still a challenge for the food industry. In this study, pasta was produced from fenugreek (FF), chickpea (CPF) and tiger nut (TNF) flours. CPF and FF are interesting for a balanced contribution of soluble and insoluble fibre by combining the health benefits of each type of fibre that promotes health. TNF, also rich in insoluble fibre, can provide additional healthy properties. The partial substitution of TNF for FF (0, 2.5, 5, 7.5 and 10% *w*/*w* solids) was assessed, and the relation linking chemical composition, structure, cooking and rheological properties and predictive in-vitro starch digestion (eGI, expected glycaemic index) was analysed. The results revealed that FF, rich in galactomannans, not only improves the nutritional profile and lowers the eGI but also helps to naturally enhance the structure of the pasta product and, thus, cooking behaviour (higher swelling index and fewer cooking losses).

## 1. Introduction

Coeliac disease (CD) is one of the most frequent food-induced disorders, with an estimated mean prevalence of 1% of the total population [1]. To date, the only efficacious treatment for CD-affected patients is a gluten-free diet. The coeliac market is increasing, but is still limited by several factors, such as availability, prices, sensorial properties and poor variability [2] as most of these products are based on corn or rice [3]. Furthermore, it has been reported that most of these products present inferior nutritional profiles [4], and a higher glycaemic index (GI) than their gluten-containing counterparts [5,6]. CD patients are at higher risk of suffering autoimmune disorders than the general population; e.g., high incidences of type 1 diabetes mellitus [7]. Therefore, good glycaemic control is an important task for CD patients [6]. Several studies have shown that people with type 1 diabetes who consume high-fibre diets have lower blood glucose levels, while higher fibre intake may improve blood sugar, lipid and insulin levels in people with type 2 diabetes [8]. Thus a pending goal to be met by food scientists is to adequately develop high-fibre gluten-free foodstuffs. Pasta is one of the products that is most demanded by people with CD disease [5], as it is appealing to both adults and children, its shelf life is long, and it is low-cost and easily prepared. Legumes and tiger nut are gluten-free ingredients that can be used to produce gluten-free pasta of high nutritional quality and a lower GI compared to cereal grains. Legumes are rich in proteins, dietary fibres and resistant starch, and are easily available. Legumes, such as peas, field beans, lentils, field peas, split peas, faba beans or chickpea flours high in proteins, have been used to improve the nutritional value of cereal-based pasta [9]. Nevertheless, the possibility of using legume flours to produce and improve the nutritional or functional characteristics of gluten-free pasta products has been scarcely investigated. To the authors’ knowledge, no research has been conducted on gluten-free composite fresh pasta based on fenugreek (FF), chickpea (CPF) and tiger nut (TNF) flours. Of all legumes, chickpeas (*Cicer arietinum* L.) are particularly high in dietary fibre (mainly insoluble) and offer a nutritious source of plant protein (rich in lysine and arginine) and associated phytochemicals, which are often lacking in a gluten-free diet [10,11]. Moreover, chickpeas are a good source of carbohydrates, polysaccharides and oligosaccharides. Fenugreek (*Trigonella foenum-graecum* L.), one of the oldest medicinal plants native to India, northern Africa and southern Europe, has special hypoglycaemic and antihyperlipidaemic properties, as suggested by the results of preliminary animal and human trials [12]. The presence of amino acid 4-hydroxyisoleucine and its high soluble fibre content, with a considerable amount of galactomannans, may inhibit the absorption of glucose from the small intestine as a result of increased viscosity in the lumen of the intestine [13]. Fenugreek is reported to also possess antioxidant activity and is especially rich in lysine, which make it a promising ingredient for developing new products with a good protein profile. Moreover, FF with about 20% gel-forming soluble fibre (galactomannan, similar to guar gum) might also be interesting to naturally enhance the structure of the pasta product (cohesive and elastic dough) and, thus, cooking behaviour (minimal cooking losses, no stickiness, reasonable firmness after cooking, etc.). Fenugreek flour has been successfully used in bread (up to a level of 10%), biscuits (up to 15%), pizza, muffins and cakes (8%–10%), extruded products (up to 2%) and dry pasta (up to 7.5%) without compromising sensorial features [14,15]. Tiger nut (*Cyperus esculentus* L.) is a sweet brown coloured tuber whose different varieties are grown worldwide in warm and temperate regions, such as southern Europe and Africa. It is rich in carbohydrates, fibre, some minerals (potassium, phosphorus and calcium), arginine and vitamins E and C [16]. This tuber is also rich in lipids with a similar fatty acid profile to olive and hazelnut oils. Consequently, in addition to its high fibre content (mainly insoluble), this confers healthy properties to this tuber [16]. Many authors have recently shown growing interest in the potential of tiger nut as an important source of food nutrients with health properties [17,18].

The aim of this work was to investigate the effect of incorporating up to 10% (*w*/*w* dry solids) into a gluten-free formula based on TNF and CPF on: (1) chemical composition; (2) techno-functional properties, i.e., rheological properties, microstructure, cooking behaviour, colour and sensory attributes and (3) in vitro starch digestibility (eGI; expected glycaemic index).

The main hypothesis of the present research was to replace TNF with FF in gluten-free fresh pappardelle based on TNF and CPF and to ascertain if it would increase the soluble fibre content, while reducing the estimated in vitro GI value and improving both the nutritional profile and structural strength.

## 2. Materials and Methods

### 2.1. Materials

The CPF used to prepare the gluten-free pasta was obtained from the supplier Rincón del Segura, S.L. (Albacete, Spain). The TNF came from Tigernuts Traders S.L. (Valencia, Spain). The FF was supplied by Especias Pedroza (Málaga, Spain). Mineral water and eggs were purchased in a local supermarket.

### 2.2. Pasta Preparation and Experimental Design

Fresh pasta was prepared by mixing the raw materials with a constant proportion of flours (76% *w*/*w*), egg (11% *w*/*w*) and water (13% *w*/*w*). Pasta doughs were prepared in an electric cooking device (Thermomix TM-5, Vorwerk Spain M.S.L., S.C., Madrid, Spain). The solid and liquid components were weighed (PFB 300-3, Kem and SohnGmbH, Balingen, Germany) and mixed: the liquids at a low speed (set 2) for 15 s before, adding the solid raw materials at an average speed (set 4 for 45 s). The resulting blends were then kneaded in two steps lasting 10 min each (with a 10 min rest period of between both). The formed doughs were allowed to stand for 20 min at 4 °C inside a plastic bag to enable sample relaxation. Afterwards, the fresh pasta sheets (1.0 ± 0.03 mm thick) were formed by using a domestic pasta-making machine (Simplex 152 SP150, Imperia, Italy), coupled with a specific motor (A2500, Imperia, Italy). The final pasta shape (pappardelle 4.1 cm ± 0.03 mm wide and 7 ± 0.03 cm long) was obtained in the same pasta-making machine with the Duplex reginette accessory 12/44 mm (model 229, Imperia, Italy). For chemical analysis, samples were stored at −45 °C (Liebherr Mediline, LCT2325, Liebherr, Baden-Wurtemberg, Germany) for 24 h before being dried in a Lioalfa-6 Lyophyliser (Telstar, Spain) at 2600 Pa and −56.6 °C for 48 h.

Several trials were carried out considering different chickpea:tiger nut ratios (40:60, 50:50 and 60:40), and machinability and sensorial analysis was considered to select 50:50 CPF:TNF. The amounts of FF to be incorporated were established after studying the maximum amount of this flour that allowed dough machinability. Thus FF was incorporated into the basis formula (50% CPF and 50% TNF, *w*/*w* solids) by replacing TNF at the 0%, 2.5%, 5%, 7.5% and 10% replacement level (w/w solids). The obtained pasta samples are named henceforth as 0FF, 2.5FF, 5FF, 7.5FF and 10FF, respectively. All the formulations were assessed in terms of nutritional composition, water activity, in vitro starch digestibility, fundamental and empirical rheological properties colour, cooking properties, microstructure and sensory acceptance.

### 2.3. Pasta Cooking

Fresh pappardelle were cooked in deionised water at 98 °C (25 g/300 mL). The optimal cooking time was previously determined with the 0FF sample according to the American Association of Cereal Chemists approved method 16–50 [19]. To avoid evaporation losses and to maintain the initial volume at 90%, the flask was covered and boiling water was added during cooking. After 10 min (optimal cooking time), pappardelle were removed from flasks and the cooking process was immediately stopped with 50 mL of cold deionised water. Finally, pasta samples were drained for 2 min and immediately analysed. The cooking trial was done in triplicate for each pasta formulation.

### 2.4. Chemical Composition of Raw Materials and Uncooked and Cooked Pasta

Flours, uncooked (UC) and cooked (C) pasta were analysed for their moisture content, protein, fat and ash according to the methods approved by the American Association of Cereal Chemists [19]. Total starch was measured in flours, and the UC and C samples with the K-TSTA enzyme kit (Megazyme Ltd., Bray, Ireland) based on the Association of Official Agricultural Chemists (AOAC) 996.11 method [20]. Total, soluble and insoluble fibre contents were determined in the flours and cooked pasta according to the AOAC 985.29 method [19] with the K-TDFR enzyme kit (Megazyme Ltd., Bray, Ireland). For the UC pasta, the fibre values were calculated by considering the experimental values for the raw materials and the proportion of these flours used in each formula. All the measurements were taken in triplicate.

### 2.5. Water Activity of Uncooked and Cooked Pasta

The water activity (a_w_) of the UC and C pasta was measured in duplicate in the AquaLab Series 4 TEV equipment (Decagon Devices, Inc., Lab-Ferrer, Lérida, Spain, CX-1, sensitivity 0.001).

### 2.6. Glycaemic Index—In Vitro Starch Digestibility of Cooked Pasta

The glycaemic index (GI) analysis was carried out based on the procedure described by [5], with minor modifications [21]. The cooked samples were crushed by a grinder (MC3001, Moulinex, Groupe SebIberia, Barcelona, Spain) for 10 s simulate chewing. To30 mL tubes, the following were added: 0.8 g of the chewed sample, 5 mL of a solution (5 mg/mL) of pepsin-HCl (Sigma-Aldrich^®^, Madrid, Spain) and 15 glass beads (4 mm in diameter). Tubes were shaken for 30 min at 60 rpm at 37 °C in a shaking bath (mod 6032011, JP Selecta S.A., Unitronic, Barcelona, Spain), where beads allowed stomach digestion conditions to be simulated (peristaltic movements). Afterwards, 12 mL of 0.1 M sodium acetate buffer (Sigma-Aldrich^®^, Madrid, Spain) were added to adjust the pH to 5.2. Then 5 mL of an enzyme mixture (7.000 U/mL) was added as well: invertase (I-9274), pancreatin (P-7545) and amyloglucosidase (A-7095) (all from Sigma-Aldrich^®^, Madrid, Spain). Tubes were re-incubated in the bath with shaking under the same conditions (37 °C, 60 rpm) and samples were extracted at different times: 0, 15, 30, 60, 10, 120 and 180 min. Immediately after each time, samples were submerged in ice to stop enzymatic activity and were then centrifuged at 4 °C, 5 min and 380 rpm (5804 R centrifuge, Eppendorf, Germany). Aliquots of 0.5 mL of the supernatant of each sample were taken and 0.5 mL of pure ethanol (Sigma-Aldrich^®^, Madrid, Spain) was added to ensure the total inactivation of enzymes. To determine the glucose concentration in each sample, an enzymatic colourimetric method with glucose oxidase (GODPOD 4058, Giesse Diagnostic SNC, Rome, Italy) was followed, using a spectrophotometer (Helios Zeta model, Thermo Fisher Scientific, Waltham, MA, USA). The proportion of hydrolysed starch was calculated at each time considering a factor of 0.9 to convert mono-to polysaccharide [5,22]. The rate of starch digestion was expressed as the percentage of TS hydrolysed at different times (0, 15, 30, 60, 100, 120 and 180 min). A first-order exponential model previously used by [22] and modified by [23] was applied on the obtained data to calculate the area under the hydrolysis curve (AUC) (0–180 min) of each gluten-free pasta. The hydrolysis index (HI) was calculated as the relation between the AUC for each pasta sample and the AUC for a reference food, white bread, which was also analysed and used as a reference [5,19]. From the HI obtained in vitro, the eGI value was then calculated using the empiric formula proposed by [24]: eGI = 8.198 + 0.862·HI. To ensure that the described methodology was correct, the eGI of the pasta based on wheat semolina was determined and corroborated with bibliographic data [2,22].

### 2.7. Rheological Properties of Dough and Pasta: Fundamental and Empirical Properties

#### 2.7.1. Dynamic Oscillatory Properties

A RheoStress rheometer (RS1-Thermo Haake, Karlsruhe, Germany), with 160 serrated parallel plate geometry surfaces (60 mm diameter, 1 mm gap), was used to perform the oscillatory tests on the pasta doughs (UC). Excess dough was carefully removed, and a thin layer of mineral oil was applied to cover the exposed sample surfaces during measurements. Before measurements, dough was allowed to stand for 5 min to allow relaxation. The analysis temperature was 20 °C. The linear viscoelastic region (LVR) was identified and established for each dough by means of stress sweep tests from 5 to 900 Pa at 1 Hz. Frequency sweeps were carried out from 10 to 0.1 Hz in the LVR (between 45 and 68 Pa, depending on the pasta sample).

#### 2.7.2. Empirical Rheological Tests: TPA and Firmness

The texture profile analysis (TPA) of the pasta doughs (UC) was performed in a TA.XT2 Texture Analyser (Stable Micro Systems, Godalming, Surrey, UK) according to the American Association of Cereal Chemists (AACC) Method 16-50 [24]. The TPA analysis provides a measurement of the textural characteristics of a product’s hardness, adhesiveness, resilience, cohesiveness and springiness. The test conditions were the following: 50% compression, 75 mm diameter probe (flat-end aluminium compression disc), test speed 1 mm/s, 75 s gap between compressions, 5 kg load cell, 10 mm dough thickness and 60 mm dough diameter (discs). Five replications were done per formulation and data were processed using Texture Exponent 6.1.7 (Stable Micro Systems Software).

Pasta firmness was assessed before (UC) and after cooking (C) by the AACC Method 16-50 [19], using the A/LKB-F cutting probe at a test speed of 0.17 mm/s until total sample deformation. Five replicates were considered for each pasta formulation.

### 2.8. Colour of Pasta

The colour of the UC and C pastas was determined in a spectrocolorimeter (Minolta CM-3600D) through the surface reflectance spectra between 400 and 700 nm (illuminant D65, 10° standard observer) using a white background. From the reflectance spectra, the CIEL*a*b* colour coordinates were obtained: L* (lightness), a* (redness-greenness), b* (yellowness-blueness). Measurements were taken in triplicate. Colour saturation ($C_{\mathrm{ab}}^{*}=\sqrt{a^{{*}^{2}}+b^{{*}^{2}}}$), hue angle (hab*=arctanb*a*), and total colour difference ($\Delta E^{*}=\sqrt{{{(L}_{i}^{*}{-L}_{j}^{*})}^{2}{+(a}_{i}^{*}{-a}_{j}^{*}{)}^{2}{+(b}_{i}^{*}{-b}_{j}^{*}{)}^{2}}$) between the uncooked (j) and cooked (i) pasta (ΔE*_1_), and between the samples without (i) and with FF flour (j) (ΔE*_2_), was calculated.

### 2.9. Cooking Properties

The water absorption index (WAI, g/g) was calculated from the mass and water content gain, according to the AACC Method 44-40 [19]. The water content of the UC and C pastas (x_w_, g water/g product) was determined according to the AACC Method 44-40 [19]. Five replicates of each sample were done. Cooking loss (amount of solids lost to cooking water, %CL) was determined according to the AACC Method 16-50 [19]. After the cooking process, the cooking and rinse waters were collected and evaporated to dryness in an air oven at 100 °C to constant weight, thus to obtain the weight of the residue. This test was performed in duplicate. The swelling index (SI) was expressed as the relative volume changes (cm^3^/cm^3^) between the UC and C pastas. The pappardelle measurements (thickness, width and length) were determined by a calliper (PCE-DCP 200N, PCE Ibérica S.L., Albacete, Spain). Six replicates of each sample were done.

### 2.10. Field Emission Scanning Electron Microscopy

Field emission scanning electron microscopy (FESEM) was used to observe the effect of incorporating FF on the pasta characteristics. For this purpose, a microstructural analysis was carried out on the longitudinal surface and cross-section of the UC and C pappardelle by FESEM (ULTRA 55, Carl Zeiss AG, Oberkochen, Germany). For the pasta observations, samples were fixed on copper stubs, platinum-coated and observed using an accelerating voltage of 2 kV.

### 2.11. Sensory Analysis

A sensory analysis of the gluten-free pasta was conducted in a laboratory in individual sensory booths with 40 untrained panellists. Two different tests were considered. Firstly, a sorting test, where the panellists had to classify samples according to a specified criterion, bitterness of pasta (1–5 scale). This test served to appreciate significant differences between samples based on relative bitter taste intensity. The five cooked pasta samples (around 25 g), coded with a random 3-digit code, were served immediately after cooking in plastic dishes. All the samples were presented randomly and simultaneously to each panellist. Successively, a Just-About-Right scale (JAR) was conducted. In this case, a single sample (10FF) was presented to the panellists. The following were assessed to evaluate different attributes by taking an ideal point as a reference: colour, sweetness, saltiness taste, bitterness, granularity, compactness and hardness. This test served to optimise the elements present in food and it was combined with a hedonic scale of global acceptance. The interpretation of the results was performed statistically as indicated in the standards.

### 2.12. Statistical Analysis

Statistical differences were determined by a one-way analysis of variance (ANOVA) and the LSD comparison test (*p* < 0.05) by using the Statgraphics Plus software, version 5.1. (StatPoint Technologies, Inc., Warrenton, VA, USA) to evaluate the effects of incorporating fenugreek incorporation on the measured parameters. Pearson’s correlation was also carried out to assess any significant correlations at *p* ≤ 0.05.

## 3. Results and Discussion

### 3.1. Chemical Composition and Water Activity (aw)

Table 1 shows the chemical composition of the flours used to prepare the gluten-free pasta. Similar results have been reported for CPF [25], TNF [18,26] and FF [14]. As expected, protein content was significantly higher (*p* < 0.05) in CPF and FF than in TFN. Therefore, the incorporation of these flours into the formulation of pastas would allow a product with a higher protein content to be obtained.

TNF, however gave a significantly higher lipid content (*p* < 0.05) which, as seen in previous studies, was rich in unsaturated fatty acids, especially oleic acid [26]. CPF was the only flour to present significantly higher starch content (*p* < 0.05), followed by TNF and well behind by FF. It is also worth mentioning the significantly higher content (*p* < 0.05) of FF total dietary fibre (TDF), especially due to substantial soluble fibre contribution. This high soluble dietary fibre content (SDF) could lead to a lower glycaemic response [27]. Moreover, insoluble dietary fibre (IDF) is predominant in TNF and CPF, which may lead to improvements in the intestinal transit [28].

The chemical composition of flours influenced that found in the obtained gluten-free pasta. The partial replacement of TFN with FF in different proportions gave rise to formulations with distinct nutritional and functional properties. Table 2 shows that the larger the amount of FF in pasta, the higher the protein and soluble fibre contents, and the lower the fat content in both the UC and C pasta samples. Only slight variation was observed in starch and minerals.

During cooking, an expected increase in moisture content (*p* < 0.05) and a decrease in dietary soluble fibre (around 2%) occurred. All the assayed formulations were declared as “high-fibre content” products as the total dietary fibre exceeded 6 g/100 g, which is in accordance with European Regulation 1924/2006 [29]. No significant differences in water activity were observed among the pasta formulations either before (values ranging between 0.911 ± 0.003 and 0.917 ± 0.002) or after (values ranging from 0.993 ± 0.003 to 0.9953 ± 0.0005) cooking.

### 3.2. In Vitro Digestion of the Cooked Pasta

The eGI obtained after the in vitro enzymatic digestion of each pasta formulation is shown in Table 3. The gluten-free pasta formulations based on CPF, TFN and FF obtained an eGI of under 55 in all cases and can, therefore, be classified as low GI food [5]. However, it was noteworthy that FF percentages over 5% (g/100 g solids) led to a significantly lower eGI (*p* < 0.05), with 10FF sample presenting a glycaemic response of 36 ± 2. The incorporation of FF into snacks [14] and different breads [30] also gave a lower eGI. The hypoglycaemic activity of FF has been mainly attributed to its high content in dietary fibre and saponins, and it is able to slow down the enzymatic digestion of carbohydrates and reduce the gastrointestinal absorption of glucose [31]. Previous research has illustrated the potential of lowering glycaemic response to foods by incorporating different fibre fractions, especially soluble fibre [32]. The modifying effect of dietary fibres in at product’s viscosity as a result of its high water-binding capacity has been shown to affect the gastric emptying, transition time and intestinal absorption of nutrients and, thus, leads to reduced glycaemic response [8]. Thus a negative correlation coefficient was found between the eGI and SDF (*r* = −0.80; *p* ≤ 0.05) of the cooked pasta. Moreover, the possible presence of phytic acid (in both legumes) and polyphenols in FF, with the capacity to reduce enzymatic digestibility [33], could have helped to lower the eGI.

**Figure 1:**
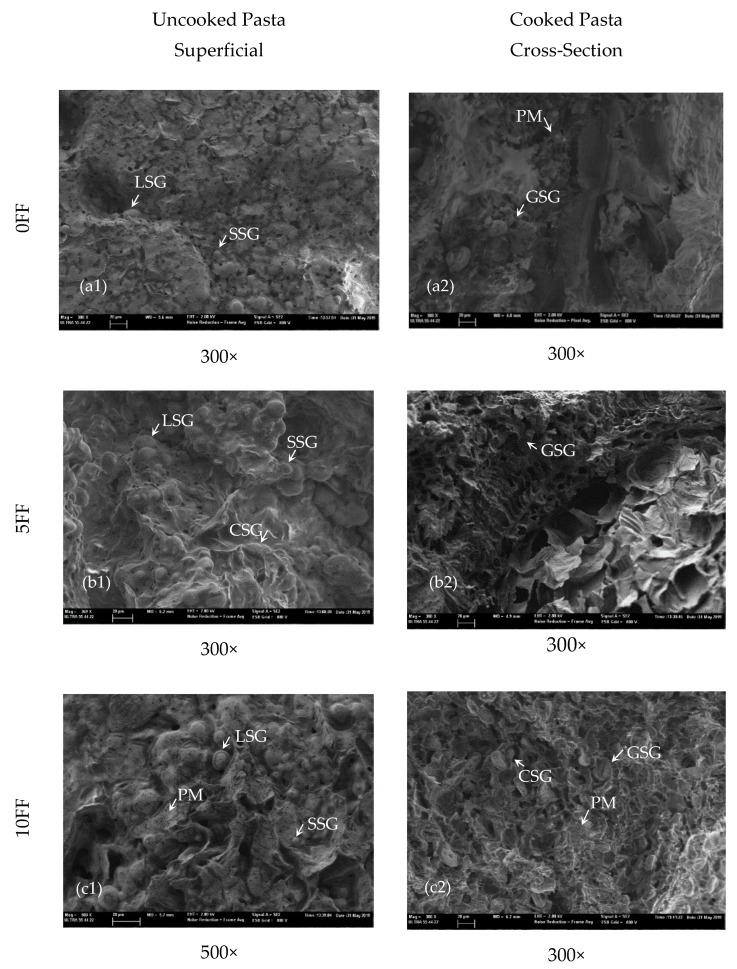
SEM observations of the 0FF, 5FF and 10FF pappardelle (magnification 300 and 500×): (**a1**–**c1**) uncooked samples; (**a2**–**c2**) cooked samples. LSG–large starch granules; SSG—small starch granules; CSG—coated starch granule; GSG—gelatinised starch granule; PM—protein matrix.

The amount of fibre, protein, starch and fat in a product, or their physical structure, are factors that govern the rate and extent of carbohydrate digestion. It has been reported that both fibre and fat can play a certain protective role during cooking by lowering the degree of starch gelatinisation and, therefore, limiting the accessibility of enzymes to starch granules once again [34]. In fact, a positive correlation coefficient was found between the fat content (it lowers as the proportion of FF is increases) and the eGI (*r* = 0.903; *p* ≤ 0.05). A positive correlation between the starch content of cooked pasta and the eGI was herein observed (*r* = 0.731; *p* ≤ 0.05). The structure of food plays a key role in the digestibility of nutrients [33]. During pasta processing, the way in which proteins encapsulate starch and how amylose lipid complexes are formed may limit the extent of starch gelatinisation during cooking and might, therefore, lower the rate at which sugar is released during in vitro digestion [34]. In this study, the partial substitution of TNF for FF led to a relatively increased protein content in pasta, which might contribute to higher protein network integrity. A negative correlation coefficient was found between the protein content of the cooked pasta (*r* = −0.752; p ≤ 0.05) and the eGI. Furthermore, in the surface micrographs of the 5 and 10 FF raw pappardelle (Figure 1), both large and small starch granules were embedded in a thick amorphous matrix. The mucilage of hydrated galactomannans (present in a considerable amount in FF) has the potential to function as a viscosity builder, and starch granules appear to be coated by gums. The results about pastas’ mechanical properties (shown below) actually evidenced a stronger denser structure when FF was present. The cross-section observations of the cooked pappardelle (Figure 1) revealed a thick sheet-like structure, probably owing to the large amount of gums, where very faint outlines of a few starch granules are visible in the 10FF sample. For 5FF, the gelatinised starch granules lost their shape.

### 3.3. Rheological Properties of Dough and Cooked Pasta

#### Dynamic Oscillatory Properties

**Figure 2:**
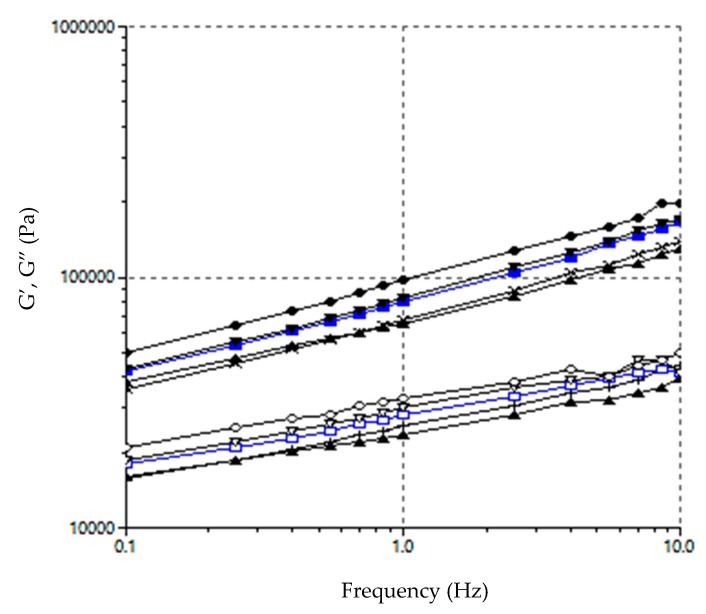
Effect of fenugreek flour (FF) addition on mechanical spectra (where G′ is represented by solid symbols and lines and G″ by open symbols and discontinuous lines). ∆ (0FF); × (2.5FF); □ (5FF); (7.5FF), ο (10FF).

Figure 2 show an example of the mechanical spectra for each pasta sample. Rheological parameters (G′ and G″) obtained at a frequency of 1 Hz are summarised in Table 4.

Table 4 shows that the storage modulus values (G′) are significantly higher (*p* < 0.05)—in all cases—than those of the loss modulus (G”) (tan δ < 1), which reveals the more elastic and less viscous nature (more solid nature) of the studied doughs. Similar results have been observed in pasta made with rice [35] and corn [36], where the loss modulus (G″ values were between 5- and 10-fold lower than the storage modulus (G′) values, and G” was lowered as frequency increased. This is indicative of a system acting more elastically [35]. This elastic behaviour is characteristic of a highly structured material, where the storage modulus is always higher than the loss modulus over the entire studied frequency range [36].

It is also possible to see that these values progressively increased with the amount of FF in dough. Thus it would seem that more protein and soluble fibre were present (mainly galactomannan), and with a high water retention capacity (as previously mentioned), which would influence the rheological behaviour of doughs in which a more cohesive and dense structure formed in these cases. In fact strong positive correlations were found between G′ and G″ with protein content (*r* = 0.96 and 0.98, respectively) and fibre (*r* = 0.96 and 0.99, respectively) at the 95% confidence level. A negative correlation between these parameters and fat content was also observed (*r* = −0.99 and 0.97, respectively; *p* ≤ 0.05). The dependence of both moduli (G′ and G″) on angular frequency (ω) can be explained by the parameters (exponents a and b) obtained by Equations 1 and 2 [37] (Table 5).

(1) $$G^{\prime }\left(\omega \right)\;=\;K^{\prime }\cdot \omega^{a},$$

(2) $$G^{\prime \prime }\left(\omega \right)\;=\;K^{\prime \prime }\cdot \omega^{b},$$

Low values of the exponents a and b, near zero, mean, respectively, that the storage modulus, G′ or the loss modulus, G″, are not dependent on the frequency. Exponent a was always higher than exponent b, which reflects a stronger dependence on the frequency of elastic moduli than the viscous one. The exponent b values of came close to 0 in all cases, which demonstrates the gel-type structure (semi-solid nature) of the studied doughs. The presence of FF brought about a significant decrease in parameter b, and the value of 10FF came closer to 0, which means less dependence of G″ on frequency. Once again, the presence of hydrated galactomannan could be responsible because the structure was more gel-like.

The effect of FF on the empirical rheological properties of the UC and C pappardelle is observed in Table 6. TPA parameters corresponds to compression tests while firmness values are obtained from a cutting test. The TPA data revealed significant differences (*p* < 0.05) between the samples with and without FF for as regards to hardness, adhesiveness, resilience, cohesiveness and springiness. This would appear to be an increasing trend of these parameters when more FF was incorporated. Greater cohesiveness and springiness could result from a better formed dough structure due to the higher protein content of such doughs (as previously mentioned), and also to certain soluble fibre hydration (galactomannan), which could contribute to act as a filler and confer the product a gel-like structure. The presence of insoluble fibre could be responsible for greater dough hardness. Positive correlations were found among the parameters of hardness, cohesiveness, springiness, resilience and fracture force with protein content (*r* = 0.994, 0.926, 0.934, 0.957, 0.894, respectively) and fibre (*r* = 0.982; 0.945; 0.956; 0.938; 0.942, respectively) at the 95% confidence level. Clearer firmness results were obtained in the UC samples, where the increase in FF led to higher values for this parameter. This result has been also observed in other studies, in which pasta was prepared with FF substitutions [13]. Once again, the bigger amount of fibre contained in these pastas could explain this result [38]. Likewise, it has been reported that fenugreek galactomannans can form films and act as gelling agents, adhesives and binders [13]. These results indicate that textural properties improved when FF was incorporated into the formulation. Otherwise, starch gelatinisation and protein coagulation phenomena, which take place during cooking, could responsible for the observed homogenisation of firmness (no a clear trend was seen in this case with increased FF).

### 3.4. Colour of the Uncooked and Cooked Pastas

Table 7 show the CIEL* a* b*, C_ab_* and h_ab_* values for the UC and C pastas. The pasta without FF, based on TNF and CPF, displayed the characteristic dark brown tones of TNF. As the FF content increased, the uncooked pappardelle became darker (lower L*) and redder (increase in the a* coordinate). This was evidenced by the increasing colour differences values (ΔE*_1_). Generally, a visible difference between samples is considered for ΔE values above 2 [39], so these colour differences were high enough to be appreciated when over 5% (*w*/*w*) FF was included, as the sensory evaluation confirmed. These tones can be attributed to the content of carotenoids and other pigments of FF [40]. The reduction in luminosity with amount of FF was less evident in the cooked pasta because water absorption during cooking. The lower values of coordinates a* and b* in the cooked samples can be explained by the degradation and dissolution of pigments in cooking water [41]. Similar results have been reported in pastas supplemented with different legumes [42]. Nevertheless, the results revealed that colour differences between the UC and C pastas ΔE*_2_) diminished as the amount of FF rose. This fact could be related to the fewer cooking losses found for the pasta that contained more FF (as described below).

### 3.5. Cooking Properties

The WAI provides information on water-absorption capacity during the cooking process, with significant differences (*p* < 0.05) between the sample without FF and that with the highest percentage, 10FF (Table 8). More fibre could increase the capacity to retain water [34], and the protein matrix may be interrupted, which would promote water absorption [43]. However, the effect on the SI was much more evident when increasing the amount of FF in the pasta formulation. Positive correlations were found between this parameter and SDF (*r* = 0.96) and IDF (*r* = 0.924) of the cooked pasta, and negative ones with fat content (*r* = −0.916), at the, 95% confidence level, hence fibre would influence water absorption and retention capacity. Cooking losses are a decisive measure of pasta quality. Pasta is generally considered to be of good quality when these losses are below 10% [9]. The values of this parameter went below 10% and, although the analysis of variance (ANOVA) did not present any significant differences, a trend appeared in which the pasta with higher percentages of FF showed fewer losses. The analysis of these parameters revealed that the samples with the most FF led to better cooking behaviour. This could be due, once again, to the presence of galactomannans in FF, which are capable of forming films and acting as gelling agents, adhesives and binders [13]. As this trend was consistent with the results obtained for the mechanical properties, we can state that the addition of FF gave rise to harder and cohesive pappardelle and, thus, to fewer losses during cooking.

### 3.6. Sensory Analysis

It has been reported that seed testa of FF provide a bitter taste [13] and, therefore, the sensory study of pasta by consumers is essential to know its acceptance. In this study, the sensory analysis of all gluten-free pasta samples revealed that the 40 panellists appreciated significant differences at a 99% level between samples. This was statistically calculated with the Friedman Test. Once the significant differences had been demonstrated, it was possible to identify the pairs of samples that significantly differed from one another using an analogue Fisher’s minimum significant difference. The 0FF sample differed significantly from the 5, 7.5 and 10FF samples, but not from the 2.5FF one. Significant differences were also observed between samples 2.5FF and 10FF. However, none of the other samples presented significant differences, so it was not possible to establish a clear order of percentages used for this flour.

**Figure 3:**
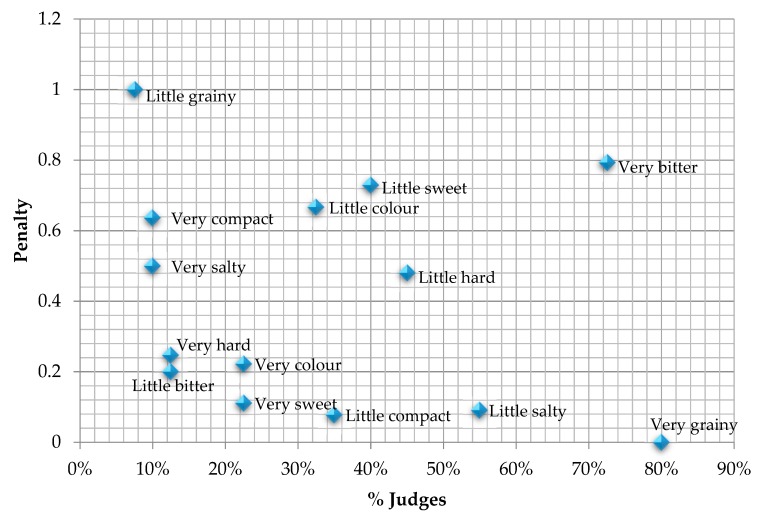
Graph of the penalties of the studied attributes on the ideal point scale.

By means of the Just-About-Right scale test (JAR), it was possible to determine if the attributes of gluten-free pasta with a lower eGI (10FF sample) were well optimised. To analyse the data, a penalty analysis was used, a method that allows to see if in fact an attribute is above or below its ideal point in relation to the global acceptance score. Figure 3 shows the preferences map of the studied attributes. As the overall acceptance of the product was 3.4 out of 7, attributes were not correctly optimised. As overall acceptance was low, no very high penalties were found and, therefore, the evaluation was made according to the percentage of judges. This indicated that the attributes that would need improving lowering their intensity, namely bitterness and granularity. Jyotsna et al. [13] also observed that the pasta with a 10% percentage of substitution obtained low scores given its persistent bitter taste. Other authors [44] have also found that due to the distinct bitter taste, inclusion of fenugreek flour was not acceptable at levels more than 2% in extruded chickpea based products. Some interesting alternatives to be studied for reducing this bitterness are soaking or soft roasting of fenugreek seeds before milling, and the inclusion of debittered fenugreek polysaccharide instead of fenugreek flour.

## 4. Conclusions

The use of CPF, TNF and FF allowed gluten-free high-fibre fresh pasta to be developed. The incorporation of FF into formulations improved the nutritional profile, and pasta had higher protein content which gave rise to a balanced contribution of soluble and insoluble fibre along with the health benefits that each fibre type promotes. The analysis of the eGI revealed that the formulations with higher FF percentages (10% *w*/*w*) had the lowest glycaemic response, probably caused by higher fibre content (especially soluble) and a denser structural network, which slows down starch enzymatic digestion. Pasta texture also improved when FF was added, which reveals that galactomannans can act as structural binders. This better structure resulted in fewer losses during cooking and also in, higher WAIs and SI. Fenugreek pigments give a more reddish pasta, but consumers did not penalise this attribute. Instead, bitterness was detected in all cases, and became especially intense over 5% FF. Very promising results were obtained from the nutritional, glycaemic response and cooking behaviour points of view, which thus extend the available supply of products to a specific population sector (coeliac and/or diabetic). Reducing the bitterness sensation to the mouth is recommended to achieve more sensory acceptance.

## Figures and Tables

**Table 1:** Proximate chemical composition of CPF, TNF and FF (g/100 g).

	CPF	TNF	FF
Water	9.33 ± 0.01 ^c^	6.4 ± 0.2 ^a^	7.96 ± 0.08 ^b^
Proteins	23.70 ± 0.09 ^c^	3.7 ± 1.5 ^a^	20.7 ± 0.3 ^b^
Fat	4.31 ± 0.03 ^a^	21.44 ± 0.06 ^c^	5.31 ± 0.05 ^b^
Ashes	3.2984 ± 0.0007 ^b^	2.516 ± 0.004 ^a^	4.3685 ± 0.0004 ^c^
Dietary Fibre			
SDF	6 ± 3 ^a^	3.5 ± 1.3 ^a^	25 ± 2 ^b^
IDF	23.9 ± 0.6 ^b^	16.6 ± 0.8 ^a^	23 ± 3 ^b^
Total	30 ± 3 ^a^	20 ± 2 ^a^	48 ± 5 ^b^
Total starch	33.6 ± 1.5 ^c^	18 ± 4 ^b^	0.4 ± 0.2 ^a^

Values are reported as means ± standard deviation of three replicates. ^a–c^ The means with different superscripts within rows significantly differ (*p* < 0.05). CPF: chickpea flour; TNF: tiger nut flour; FF: fenugreek flour; SDF: soluble dietary fibre; IDF: insoluble dietary fibre.

**Table 2:** Chemical characterisation of the different processed gluten-free pastas before and after cooking (g/100 g, dry basis). Numbers in the sample’s code indicate the percentage level of tiger nut flour replaced by fenugreek flour

	Before Cooking				
	0FF	2.5FF	5FF	7.5FF	10FF
Water^1^	25.7 *±* 0.2 ^a.A^	27.7 *±* 0.5 ^c.A^	27.656 *±* 0.101 ^c.A^	26.4 *±* 0.2 ^b.A^	27.2 *±* 0.3 ^c.A^
Protein	12.12 *±* 0.06 ^a.A^	12.589 *±* 0.012 ^b.A^	13.6 *±* 0.2 ^c.A^	13.8 *±* 0.2 ^c.A^	14.45 *±* 0.19 ^d.A^
Fat	14.42 *±* 0.07 ^d.A^	14.234 *±* 0.113 ^c.A^	13.447 *±* 0.014 ^b.A^	13.30 *±* 0.04 ^b.A^	12.50 *±* 0.03 ^a.A^
Ash	2.6571 *±* 0.0003 ^a.A^	2.8366 *±* 0.0008 ^ab.A^	2.7927 *±* 0.0013 ^ab.A^	2.8849 *±* 0.0008 ^b.A^	2.9134 *±* 0.0004 ^b.A^
Dietary Fibre					
SDF	4.86 *	5.55 *	6.10 *	6.54 *	7.16 *
IDF	20.76 *	21.51 *	21.66 *	21.47 *	21.87 *
Total	25.61 *	27.06 *	27.76 *	28.00 *	29.02 *
Total starch	29.3 *±* 0.5 ^b.A^	28.5 *±* 0.7 ^ab.A^	29 *±* 2 ^ab.A^	27.7 *±* 0.4 ^a.A^	27.9 *±* 0.3 ^ab.A^
	**After Cooking**				
	**0FF**	**2.5FF**	**5FF**	**7.5FF**	**10FF**
Water^1^	58 *±* 2 ^a.B^	60.7 *±* 0.5 ^b.B^	57.83 *±* 1.09 ^a.B^	60.83 *±* 1.12 ^b.B^	58.9 *±* 1.3 ^ab.B^
Proteins	13.04 *±* 0.02 ^b.B^	13.4 *±* 0.2 ^c.B^	10.98 *±* 0.14 ^a.B^	14.79 *±* 0.13 ^d.B^	14.67 *±* 0.03 ^d.A^
Fat	15.89 *±* 0.04 ^c.B^	15.2 *±* 0.4 ^b.A^	14.85 *±* 0.04 ^b.B^	13.18 *±* 0.02 ^a.A^	12.80 *±* 0.04 ^a.B^
Ashes	2.419 *±* 0.003 ^a.A^	2.464 *±* 0.005 ^a.A^	2.242 *±* 0.002 ^a.A^	2.614 *±* 0.004 ^a.A^	2.680 *±* 0.003 ^a.A^
Fibre					
SDF	3 *±* 2 ^a^	3.5 *±* 0.4 ^a^	4.2 *±* 1.2 ^a^	4.3 *±* 0.6 ^a^	4.9 *±* 1.3 ^a^
IDF	19.2 *±* 1.3 ^a^	20 *±* 3 ^a^	22 *±* 5 ^a^	24 *±* 4 ^a^	22.4 *±* 1.8 ^a^
Total	22.31 *±* 3.69 ^a^	23 *±* 3 ^a^	26 *±* 6 ^a^	29 *±* 4 ^a^	27.3 *±* 0.6 ^a^
Total starch	32.6 *±* 0.9 ^c.B^	30.6 *±* 1.3 ^bc.B^	29 *±* 2 ^ab.A^	28.4 *±* 1.5 ^ab.A^	27.8 *±* 1.7 ^a.A^

Values are reported as means ± standard deviation of three replicates. ^a–d^ The means with different superscripts within rows significantly differ (*p* < 0.05). ^A,B^ The means with different superscripts within columns and for each parameter significantly differ (*p* < 0.05). SDF: soluble dietary fibre; IDF: insoluble dietary fibre. ^1^ Water content expressed as g/100 g product. * Data calculated from the raw material data.

**Table 3:** Results obtained for the expected glycaemic index (eGI).

Formulation	eGI
0FF	46.9 ± 0.9 ^c^
2.5FF	44.6 ± 1.4 ^bc^
5FF	46.56 ± 0.97 ^c^
7.5FF	41.62 ± 1.12 ^b^
10FF	36.3 ± 1.7 ^a^

Values are reported as means ±standard deviation of three replicates. ^a–c^ The means with different superscripts within rows significantly differ (*p* < 0.05).

**Table 4:** Dynamic oscillatory properties at a frequency of 1 Hz: elastic storage modulus (G′) and viscous loss modulus (G″) of the UC pasta blends.

Formulation	G′ (Pa)	G″ (Pa)
0FF	64,736 ± 2155 ^c^	23,500 ± 170 ^c^
2.5FF	66,908 ± 2739 ^c^	24,688 ± 1228 ^c^
5FF	80,263 ± 1794 ^b^	27,430 ± 800 ^b^
7.5FF	85,373 ± 5016 ^b^	30,110 ± 1432 ^a^
10FF	105,236 ± 7217 ^a^	31,900 ± 809 ^a^

Values are reported as means ±standard deviation of three replicates. ^a–c^ The means with different superscripts within rows significantly differ (*p* < 0.05).

**Table 5:** Parameters obtained for the fitted Equations (1) and (2) for the elastic-G′- and viscous-G″- moduli affected by frequency (ω).

Formulation	$G^{\prime }\left(\omega \right)=K^{\prime }\cdot \;\omega^{a}$		$G^{\prime \prime }\left(\omega \right)=K^{\prime \prime }\cdot \;\omega^{b}$	
	K’ [Pa s^n^]	a	K″ [Pa s^n^]	b
0FF	66,259 ± 2551 ^c^	0.279 ± 0.015 ^ab^	23,684 ± 301 ^d^	0.198 ± 0.015 ^ab^
2.5FF	67,653 ± 3021 ^c^	0.279 ± 0.006 ^a^	24,570 ± 1310 ^d^	0.22 ± 0.01 ^a^
5FF	81,638 ± 2119 ^b^	0.293 ± 0.008 ^ab^	27,145 ± 650 ^c^	0.192 ± 0.012 ^b^
7.5FF	86,431 ± 4996 ^b^	0.2923 ± 0.0114 ^ab^	29,472 ± 1190 ^b^	0.193 ± 0.008 ^b^
10FF	106,227 ± 7809 ^a^	0.27 ± 0.02 ^b^	31,504 ± 499 ^a^	0.156 ± 0.019 ^c^

Values are reported as means ±standard deviation of three replicates. ^a–d^ Within columns, values with the same following letter do not significantly differ from one another (*p* > 0.05).

**Table 6:** Empirical rheological properties: TPA parameters of the uncooked (UC) doughs and firmness of the UC and cooked (C) gluten-free pasta.

Formulation	Hardness (N)	Adhesiveness (N·s)	Resilience	Cohesiveness	Springiness	Firmness (N)	
						UC	C
0FF	172 ± 14 ^a^	−0.34 ± 0.09 ^b^	0.116 ± 0.014 ^a^	0.243 ± 0.019 ^a^	0.24 ± 0.02 ^a^	21 ± 3 ^a. A^	28 ± 4 ^a b. A^
2.5FF	215 ± 30 ^b^	−0.61 ± 0.17 ^a b^	0.14 ± 0.02 ^a b^	0.281 ± 0.007 ^b^	0.28 ± 0.04 ^a b^	25 ± 3 ^a. A^	25.3 ± 1.2 ^a. A^
5FF	267 ± 7 ^c^	−0.8 ± 0.3 ^a b^	0.154 ± 0.012 ^b c^	0.287 ± 0.009 ^b^	0.308 ± 0.008 ^b^	25 ± 3 ^a. A^	31.1 ± 0.7 ^b. B^
7.5FF	279 ± 14 ^c^	−1.1 ± 0.7 ^a^	0.15 ± 0.02 ^b c^	0.29 ± 0.03 ^b^	0.34 ± 0.03 ^b^	31.3 ± 0.8 ^b. B^	27 ± 2 ^a b. A^
10FF	313 ± 10 ^d^	−1.13 ± 0.17 ^a^	0.17 ± 0.02 ^c^	0.33 ± 0.02 ^c^	0.44 ± 0.09 ^c^	36.4 ± 1.9 ^c. B^	29 ± 3 ^a b. A^

Values are reported as means ±standard deviation of three replicates. ^a–^^d^ The means with different superscripts within columns significantly differ (*p* < 0.05), ^A,B^ The means with different superscripts in firmness within columns significantly differ (*p* < 0.05).

**Table 7:** Colour profile (colour coordinates L*, a* and b*, chrome (C*_ab_) and hue angle (h*_ab_)) of the uncooked and cooked pasta and colour difference between the sample without fenugreek and the rest with the different percentages of substitution (ΔE*_1_), and between UC and C samples (ΔE*_2_).

	Formulation	L*	a*	b*	C*_ab_	h*_ab_	∆E*_1_	∆E*_2_
UC	0FF	48.6 ± 0.2 ^b^	8.72 ± 0.02 ^c^	26.24 ± 0.11 ^d^	26.652 ± 0.102 ^d^	71.62 ± 0.08 ^c^	-	-
	2.5FF	48.2 ± 0.2 ^b^	8.83 ± 0.05 ^cd^	26.9 ± 0.2 ^d^	28.3 ± 0.2 ^d^	71.819 ± 0.109 ^c^	0.80 ± 0.12 ^a^	-
	5FF	46.8 ± 0.2 ^a^	8.99 ± 0.12 ^cd^	26.48 ± 0.15 ^d^	27.96 ± 0.13 ^d^	71.2 ± 0.3 ^bc^	1.9 ± 0.5 ^ab^	-
	7.5FF	46.3 ± 0.3 ^a^	9.47 ± 0.08 ^e^	26.7 ± 0.5 ^d^	28.3 ± 0.5 ^d^	70.4 ± 0.3 ^a^	2.5 ± 0.2 ^b^	-
	10FF	46.3 ± 0.3 ^a^	9.2 ± 0.3 ^de^	26.1 ± 0.5 ^d^	27.7 ± 0.5 ^d^	70.6 ± 0.4 ^ab^	2.4 ± 0.4 ^b^	-
C	0FF	60.7 ± 0.2 ^f^	4.692 ± 0.017 ^ab^	20.0 ± 0.7 ^c^	20.6 ± 0.6 ^c^	76.8 ± 0.5 ^f^	-	14.86
	2.5FF	57.1 ± 0.9 ^e^	4.4 ± 0.4 ^a^	17.9 ± 1.4 ^a^	18.4 ± 1.4 ^a^	76.13 ± 0.14 ^de^	4.4 ± 0.6 ^b^	14.42
	5FF	55.5 ± 0.6 ^d^	4.60 ± 0.09 ^ab^	17.9 ± 0.6 ^a^	18.5 ± 0.6 ^a^	75.6 ± 0.2 ^de^	5.7 ± 0.3 ^ab^	13.98
	7.5FF	54.8 ± 1.4 ^cd^	4.8 ± 0.5 ^b^	18.8 ± 0.9 ^ab^	19.39 ± 1.05 ^ab^	75.6 ± 0.8 ^d^	6.13 ± 1.16 ^ab^	13.57
	10FF	53.8 ± 1.2 ^c^	4.8 ± 0.2 ^b^	19.8 ± 0.3 ^bc^	20.3 ± 0.3 ^bc^	76.2 ± 0.5 ^ef^	6.89 ± 1.19 ^b^	11.78

Values are reported as means ± SD of three determinations. ^a–f^ The means with different superscripts within columns significantly differ (*p* < 0.05) (samples UC and C analysed separately).

**Table 8:** Cooking properties: water absorption index (WAI), swelling index (SI) and cooking losses (CL).

Formulation	WAI (g/g)	SI (cm^3^/cm^3^)	CL (%)
0FF	0.700 ± 0.018 ^ab^	0.33 ± 0.02 ^a^	7.3 ± 0.5 ^a^
2.5FF	0.71 ± 0.05 ^bc^	0.5090 ± 0.1004 ^b^	6.7 ± 0.8 ^a^
5FF	0.66 ± 0.04 ^a^	0.7 ± 0.2 ^bc^	6.252 ± 0.109 ^a^
7.5FF	0.74 ± 0.03 ^bc^	0.75 ± 0.08 ^c^	6.36 ± 0.17 ^a^
10FF	0.74 ± 0.04 ^c^	0.76 ± 0.15 ^c^	5.9 ± 0.6 ^a^

Values are reported as means ± standard deviation of three replicates. ^a-c^ The means with different superscripts within columns significantly differ (*p* < 0.05).
